# Towards precision bio(hydro)metallurgy: tailored biological systems for rare earth element recovery from dilute and secondary streams

**DOI:** 10.3389/fbioe.2026.1920659

**Published:** 2026-10-28

**Authors:** Maximilian K. Lübben, Paul N. Evans, Michael Paper, Nikolaus Stellner, Daniel Garbe, Sayari Mukherjee, Manoj Sekaran, Ramkrishna Sen, Gassan Hodaifa, Susan T. L. Harrison, Gerhard Schenk, Thomas B. Brück

**Affiliations:** 1 Werner Siemens-Chair of Synthetic Biotechnology, TUM School of Natural Sciences, Technical University of Munich, Garching, Germany; 2 TUM-AlgaeTec Center, TUM School of Engineering and Design, Technical University of Munich, Taufkirchen, Germany; 3 The Australian Centre for Ecogenomics, School of Chemistry and Molecular Biosciences, The University of Queensland, Brisbane, QLD, Australia; 4 School of Chemistry and Molecular Biosciences, The University of Queensland, Brisbane, QLD, Australia; 5 TUMCREATE Ltd., Singapore, Singapore; 6 Department of Bioscience and Biotechnology, Indian Institute of Technology Kharagpur, Kharagpur, West Bengal, India; 7 Department of Biotechnology, School of Bio Sciences and Technology, Vellore Institute of Technology, Vellore, Tamil Nadu, India; 8 Molecular Biology and Biochemical Engineering Department, Universidad Pablo de Olavide, Dos Hermanas, Sevilla, Spain; 9 Office of the Deputy Vice Chancellor for Research & Innovation, The University of Queensland, Brisbane, QLD, Australia; 10 Centre for Bioprocess Engineering Research, Department of Chemical Engineering, University of Cape Town, Cape Town, South Africa; 11 ARC Research Hub for Engineering Plants to Replace Fossil Carbon, The University of Queensland, Brisbane, QLD, Australia

**Keywords:** bio(hydro)metallurgy, biosorption, circular economy, lanthanide-binding proteins, precision biometallurgy, rare earth elements

## Abstract

Rare earth elements (REEs) are indispensable to technologies underpinning the energy transition, advanced manufacturing and digital infrastructure. However, their extraction and separation remain resource-intensive and are increasingly challenged by declining ore grades and the growing importance of dilute, chemically complex secondary resources. Biological processes offer a complementary strategy by exploiting molecular recognition mechanisms beyond conventional physicochemical separations. Biological macromolecules, cellular structures and metabolic processes create affinity-driven, three-dimensional binding environments that enable selective enrichment and recovery of REE groups, while high-resolution separation of individual lanthanides is currently achievable primarily through advanced affinity-based systems. This perspective examines emerging approaches to biological REE recovery, including bioleaching, biosorption, bioaccumulation, extracellular polymeric substances, lanthanide-binding proteins, and engineered biomaterials. We argue that their greatest near-term value lies in hybrid deployment, where biological systems act as selective solubilization, enrichment or polishing modules integrated with established hydrometallurgical operations. Particular emphasis is placed on scalable engineering solutions, including immobilized biosorbents and modular recovery systems compatible with existing metallurgical infrastructure. Realizing this potential requires a shift from proof-of-concept studies in simplified laboratory solutions toward validation in industrial leachates, rigorous assessment under realistic competitor-ion conditions, regeneration and reuse of biological materials, and integrated techno-economic and life-cycle analyses. Framing biology as a programmable platform for selective metal recognition and enrichment establishes a pathway toward hybrid recovery technologies supporting a more circular and sustainable critical mineral supply.

## 1 Introduction - why REEs need new separation paradigms

Rare earth elements (REEs) comprise the lanthanide metals together with yttrium and scandium. Their optical, catalytic and magnetic properties make them essential in permanent magnets, wind turbines, electric vehicles, light-emitting devices, batteries, polishing agents and advanced electronic systems (Jha et al., 2016; Brown et al., 2023; Vítová and Mezricky, 2024). Their strategic importance is amplified by rising demand, long lead times for mine development, limited availability of high-grade resources (Valenta et al., 2023), and the geographic concentration of mining, refining and magnet production, which increases exposure to price volatility and supply disruption (Paper et al., 2023a; Kim et al., 2026).

REE recovery is challenging because these elements rarely occur in concentrated, readily separable forms and because adjacent lanthanides exhibit highly similar trivalent chemistry, ionic radii, coordination behavior, and ligand affinities (Chen et al., 2025). Conventional purification relies on ion exchange, selective precipitation, and especially solvent extraction, in which small differences in partition coefficients are amplified through multistage mixer–settler circuits. These processes are industrially effective but resource-intensive, consuming substantial quantities of solvents, acids, water, and energy and generate complex waste streams (Ortmann et al., 2025).

The environmental and economic burden of conventional REE production is increasing with global demand. Although REE oxide production has expanded, end-of-life recycling remains extremely limited, at approximately 1% (Beylot et al., 2020). Depending on ore type and processing route, producing one ton of REEs may generate up to 2,000 tons of residues, 40–110 tons of CO_2_ emissions, and up to 1,000 tons of contaminated wastewater (Geng et al., 2023). Conventional processing combines beneficiation, roasting or digestion, leaching, and extensive solvent extraction, ion exchange, or precipitation circuits (Jha et al., 2016; Gkika et al., 2024). These routes are best established for concentrated primary feeds but are less attractive for dilute or chemically complex secondary resources, including phosphogypsum, mine tailings, coal discards, fly ash, red mud, kaolin residues, mine waters, metallurgical effluents, landfill leachates, electronic waste, spent magnets and fluorescent lamp residues (Koch et al., 2024; Joshi et al., 2025; Gkika et al., 2024).

REEs can be mobilized from these materials by acidolysis, complexolysis or redox-driven leaching (Soto-Montandon et al., 2025), yet the resulting leachates remain chemically complex and are often dominated by competing ions such as Fe, Al, Ca, Mg, sulfate and phosphate. Efficient enrichment, impurity removal and selective recovery are therefore essential. This perspective differs from existing reviews in scope and structure. Biosorption-focused overviews either pre-date the discovery of high-affinity lanthanide-binding proteins (Das and Das, 2013) or treat biosorption as a stand-alone recovery technology rather than as one module within a process sequence (Giese, 2020). Broader surveys cover bioleaching and biosorption but do not extend to cell-free affinity separation (Brown et al., 2023), whereas recent reviews emphasize bioleaching economics (Huang et al., 2025) or engineered biomaterials in isolation (Ye et al., 2024). Here, we propose an integrated cascading architecture in which solubilization, enrichment, and affinity separation operate as sequential, complementary modules, paired with explicit translational criteria for industrial deployment. The strategic importance of REEs, the associated separation challenges, and the opportunities for biological recovery are summarized in Figure 1, while translational criteria are provided in Supplementary Tables S1-S3.

**FIGURE 1:**
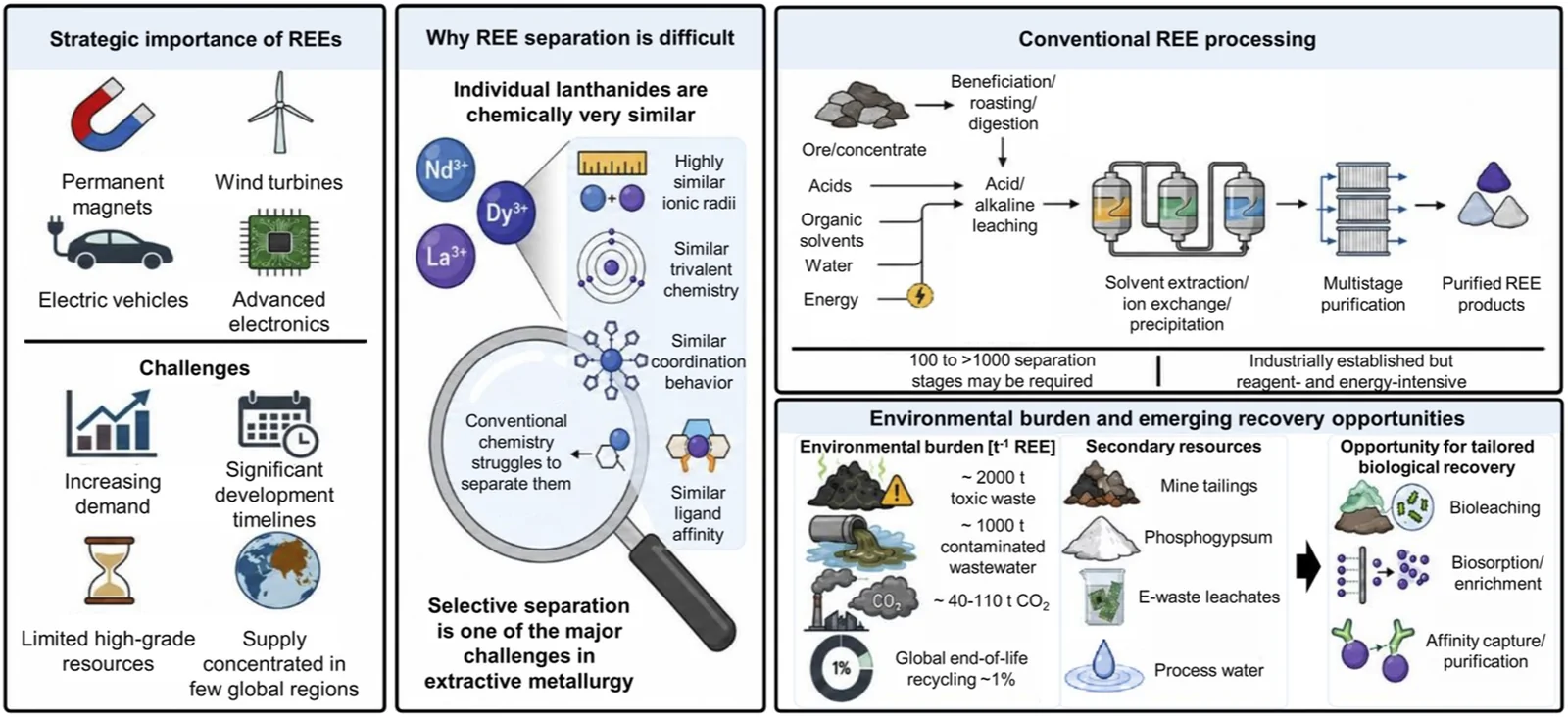
Rare Earth Element Recovery: Strategic Importance, Separation Challenges and Opportunities for Biological Recovery. The figure summarizes major REE applications and supply constraints, the chemical similarity that complicates inter-lanthanide separation, the multistage nature and environmental burden of conventional processing, and the growing opportunity to recover REEs from secondary resources using bioleaching, biosorption-based enrichment, and affinity capture. Biological systems complement conventional separation by exploiting affinity-driven recognition rather than relying exclusively on small differences in bulk physicochemical properties. Created in BioRender. Lübben, M. (2026). https://BioRender.com/rje8uae.

Alternative hydrometallurgical concepts, including greener solvent systems, magnetic-field-assisted separation, evaporation concentration, porous media, and selective ligands, are also under development but remain constrained by cost, scalability, selectivity, or integration into existing infrastructure (Ortmann et al., 2025). Biological systems offer a complementary separation logic. Macromolecules, cellular surfaces, and extracellular matrices form affinity-driven three-dimensional binding environments capable of recognizing metals under comparatively mild conditions (Dong et al., 2021). In this perspective, precision refers to the recognition mechanism rather than to a uniform separation resolution. Bioleaching and biosorption largely operate at REE-group level, whereas engineered affinity systems can resolve individual lanthanides. One example is the single-stage Nd^3+^/Dy^3+^ separation, where >98% purity has been demonstrated (Mattocks et al., 2023). Precision is therefore delivered primarily by the affinity stage and enabled by upstream solubilization and enrichment.

Classical biological REE recovery encompasses bioleaching, biosorption, and bioaccumulation (Soto-Montandon et al., 2025; Giese, 2020; Vítová and Mezricky, 2024; Joshi et al., 2025). These approaches sit within the broader concept of biomining, which historically included both metal solubilization and biosorptive concentration (Kratochvil and Volesky, 1998; Volesky and Holan, 1995; Vítová and Mezricky, 2024). Tailored bio(hydro)metallurgy extends this foundation by treating living cells, inactivated biomass, extracellular polymers, natural or engineered proteins, and biomaterials as distinct process materials selected for specific feed matrices and separation tasks. This perspective paper proposes an industrially oriented sequential processing approach in which lixiviant-producing microbial systems mobilize REEs, phototrophic or inactivated biomass provides enrichment and pre-concentration, and highly selective proteins such as lanmodulin (LanM) support final purification (Cotruvo et al., 2018).

## 2 Emergence of tailored biological REE separation systems

Biological REE recovery can be organized into three functional stages aligned with Figure 2: solubilization from solid phases (Stage 1), enrichment from dilute aqueous streams (Stage 2), and high-resolution affinity separation (Stage 3). These modules exploit natural and engineered recognition mechanisms at different selectivity levels and are designed to complement established (bio)hydrometallurgical operations. Their feed context, process function, advantages, limitations and maturity are compared in Supplementary Table S1.

**FIGURE 2:**
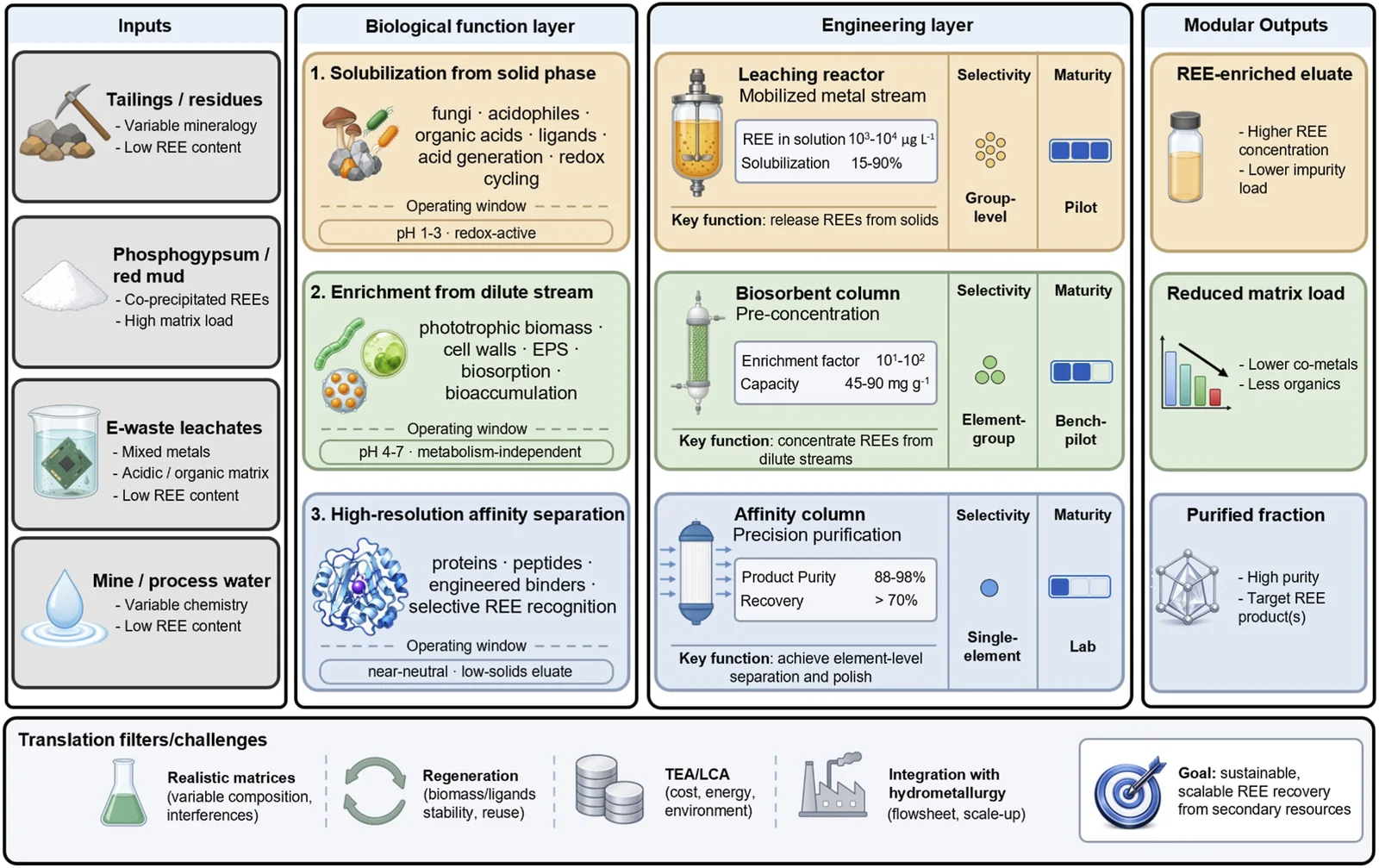
Integrated cascading bio(hydro)metallurgical framework for rare earth element recovery from dilute and heterogeneous secondary resources. Complex feedstocks, including mine tailings and residues, phosphogypsum and red mud, electronic-waste leachates and mine or process waters, are processed through three complementary stages. Stage 1 mobilizes REEs from solid matrices through microbial or metabolite-mediated leaching. Stage 2 concentrates REEs from dilute aqueous streams using phototrophic biomass, cell-wall functional groups and extracellular polymeric substances in immobilized biosorbent systems. Stage 3 enables high-resolution purification using lanthanide-binding proteins, engineered peptides and affinity materials integrated into membranes, particles or columns. Across the cascade, matrix complexity decreases and selectivity increases from group-level recovery to element-group enrichment and targeted single-element separation. Qualitative maturity indicators reflect technologies demonstrated at laboratory, bench-pilot, and pilot scales. The operating conditions and performance ranges shown are illustrative rather than prescriptive and vary with feedstock composition, matrix chemistry, organism or biomaterial, and process configuration. Bioleaching concentrations and yields are based on Joshi et al. (2025) and Rasoulnia et al. (2021); biosorption capacities on Paper et al. (2023b); preconcentration factors on Gupta et al. (2019); and affinity-based purity and recovery on Dong et al. (2021), Mattocks et al. (2023), Ye et al. (2023), and Hussain et al. (2024). Industrial translation requires validation in realistic matrices, regeneration and reuse, techno-economic and life-cycle assessment, and integration with established hydrometallurgical infrastructure. Created in BioRender. Lübben, M. (2026). https://biorender.com/jbvh8y7.

### 2.1 Stage 1 - biological solubilization from solid phases

Biological mobilization from mine tailings, coal discards, fly ash, phosphogypsum, red mud, and electronic waste can be driven by heterotrophic or chemolithoautotrophic microorganisms. Fungal heterotrophs (e.g., *Aspergillus* and *Penicillium*) mobilize metals through organic acid production, whereas chemolithoautotrophs such as *Acidithiobacillus*, *Leptospirillum*, *Sulfobacillus* and *Acidiplasma* promote sulfur and iron oxidation, resulting in acid generation and redox cycling (Rasoulnia et al., 2021; Brown et al., 2023; Soto-Montandon et al., 2025; Kölbl et al., 2022).

These routes differ mechanistically. Fungal organic acids, such as citrate and oxalate, combine acidolysis with ligand-driven complexolysis and may provide partial selectivity via carboxylate coordination at moderate pH. Chemolithoautotrophic leaching instead relies on biogenic sulfuric acid production and Fe(III)-driven redox cycling under strongly acidic conditions. This route may co-mobilize substantial Fe, Al and sulfate loads, increasing the burden on downstream purification (Rasoulnia et al., 2021).

Bioleaching is therefore best viewed as an upstream mobilization step that transfers REEs into leachates requiring subsequent enrichment and purification. Its industrial relevance increases when integrated with selective capture, regeneration, and product recovery, particularly in staged or sequential leaching concepts (Zhang et al., 2021; Li et al., 2024).

### 2.2 Stage 2 - biological enrichment from dilute aqueous streams

Industrial leachates and wastewaters are often too dilute and chemically complex for direct high-resolution separation. Biosorption provides a rapid, metabolism-independent enrichment route using inactive, pre-extracted or immobilized biomass. REE binding occurs via cell wall and extracellular polymeric substance (EPS) functional groups. Carboxyl and phosphate groups engage REEs primarily through ion exchange and surface complexation, whereas hydroxyl- and amine-containing moieties can form higher-order coordination complexes. Their relative contributions depend on the functional-group density, metal speciation, and pH (Fomina and Gadd, 2014; De Philippis et al., 2011).

Acidic leachates (pH 1-3) create a central operating trade-off: low pH maintains REE solubility but protonates binding sites and suppresses biosorption, whereas pH adjustment improves adsorption but can promote REE precipitation, particularly in phosphate-containing matrices above pH 2-3 (Kastenhofer et al., 2024). Feed conditioning should therefore be minimized and tailored to the matrix. Acid-tolerant systems offer a complementary route. Immobilized *Galdieria sulphuraria* in calcium-alginate beads removed 97.2% La^3+^, 96.2% Y^3+^ and 98.9% Sm^3+^ from acidic mining drainage (Sun et al., 2022), demonstrating that effective recovery is achievable under acidic conditions without extensive neutralization.

Phototrophic microorganisms such as cyanobacteria and microalgae present a suitable low-cost route to biosorbent production through photosynthesis. Open-pond cultivation is established and can be comparatively simple and energy-efficient in suitable climates (Brennan and Owende, 2010; Christenson and Sims, 2011; Craggs et al., 2011). Their cell walls and EPS contain carboxyl, hydroxyl, sulfate, and other binding groups (De Philippis et al., 2011; Paper et al., 2023a; Paper et al., 2023b), and together with siderophores may provide additional surface-associated metal interactions (Årstøl and Hohmann‐Marriott, 2019). Thus, the principal process advantage of phototrophs is economical biosorbent generation rather than an enhancement of the biosorption mechanism itself.

Phototrophic systems exhibit broad physiological tolerance (Paper et al., 2023a; Paper et al., 2023b), with extremophiles such as *G*. *sulphuraria* extending operation into acidic or saline conditions (Iovinella et al., 2023; Singh et al., 2023). Biomass must nevertheless be matched to feed chemistry rather than treated as a universal sorbent. Biosorption primarily enables group-level REE enrichment and pre-concentration, whereas bioaccumulation can provide additional concentration but requires active metabolism (Rocha et al., 2024; Vítová and Mezricky, 2024). Controlled desorption regenerates the sorbent and transfers REEs into smaller, more concentrated eluate suitable for downstream processing (Giese, 2020; Gupta et al., 2019; Sun et al., 2022).

### 2.3 Stage 3 - high-resolution affinity separation

The discovery of lanthanide-dependent microbial systems has reshaped understanding of the biological role of REEs. Certain methylotrophic bacteria employ lanthanides as essential cofactors in XoxF-type, pyrroloquinoline quinone-dependent methanol dehydrogenases, demonstrating specific metabolic functions for these elements (Keltjens et al., 2014). These insights have intensified interest in the mechanisms that govern lanthanide acquisition, discrimination, and regulation under dilute, environmentally relevant conditions.

A key advance is lanmodulin (LanM), a small protein with picomolar affinity and strong selectivity for trivalent lanthanides over abundant competing ions such as Ca^2+^ (Cotruvo et al., 2018; Dong et al., 2021). This selectivity distinguishes lanthanides as a group from non-lanthanide ions. The discrimination between adjacent lanthanides is a separate and considerably harder challenge that has so far required engineered variants (Mattocks et al., 2023). Additional systems, including lanpepsy (LanP), dedicated transport components, and small-molecule chelators such as the lanthanophore methylolanthanin, further highlight the diversity of evolved recognition strategies (Rocha et al., 2024; Ye et al., 2024; Hemmann et al., 2023). However, lanthanide mobilization, trafficking, storage, and homeostasis remain only partly resolved, limiting direct translation into engineered recovery systems.

Programmable recovery strategies include engineering high-affinity REE-binding proteins, displaying selective motifs on cell surfaces, overproduction of lanthanophores and other chelators, and metabolic engineering for intracellular accumulation. Cell surface display of lanthanide-binding tags on the S-layer of *Caulobacter crescentus* increased REE adsorption and specificity over competing ions and enabled citrate elution without measurable capacity loss over repeated adsorption-desorption cycles (Park et al., 2016); the approach was later applied to low-grade feedstock leachates (Park et al., 2017). Overproduction of methylolanthanin increased lanthanide adsorption and bioaccumulation in *Methylobacterium extorquens* AM1, supporting its development as a chassis for microbial lanthanide mining (Zytnick et al., 2024). At the protein level, the separation-capable LanM constructs are engineered variants rather than native variants (Mattocks et al., 2023). These examples demonstrate that programmable biological recovery is emerging in practice, although it remains at laboratory or bench scale.

Naturally evolved and engineered binders form the basis of the most selective step of the cascade: cell-free affinity systems that discriminate between lanthanides and competing ions. Purified proteins such as LanM, derived peptides, and engineered variants can be immobilized on solid supports, membranes, magnetic nanoparticles or polymer matrices (Dong et al., 2021; Ye et al., 2023; Ye et al., 2024; Hussain et al., 2024). Their independence from cellular metabolism facilitates direct integration into existing metallurgical infrastructure.

LanM-based systems already bridge biochemistry and hydrometallurgy by exploiting differences in binding and elution behavior (Dong et al., 2021). LanM-functionalized magnetic nanoparticles and recyclable protein biosorbents further demonstrate reusable, high-selectivity materials for complex matrices (Ye et al., 2023; Hussain et al., 2024). Advances in protein structure prediction and computational design may enable binders tailored to pH windows, competing ion backgrounds, or target element pairs, although *de novo* REE-binder design remains an emerging capability rather than an industrially mature technology (Caldwell et al., 2020; Kim et al., 2026).

### 2.4 Cascading biological recovery platforms

The strongest opportunity for biological REE recovery lies in cascading process architectures. Microbial or chemical leaching first mobilizes REEs from solid matrices. Biosorptive enrichment then reduces volume and matrix complexity, after which immobilized proteins, engineered peptides, or other affinity materials provide high-resolution purification.

This division of labor mirrors conventional metallurgical flowsheets, in which bulk concentration precedes high-purity refining (Jin et al., 2017; Dong et al., 2021; Ye et al., 2024). It also reduces performance burdens on individual modules: biosorbents enrich REE groups, while affinity systems operate on smaller and cleaner eluates. This architecture is therefore more realistic for industrial deployment than isolated biological unit operation. The roles of the individual platforms are summarized in Supplementary Table S1.

## 3 From waste to resource: emerging deployment opportunities

Biological REE recovery should be deployed according to process function. In chemolithotrophic and acidophilic bioleaching systems, growth and metal mobilization are coupled because metabolism directly drives solubilization (Rawlings and Johnson, 2007). In biosorption systems, biomass production can instead be decoupled from metal capture, allowing growth to be optimized separately from exposure to harsh leachates. Supplementary Figure S1 presents a deployment-oriented waste-to-resource concept, and Supplementary Table S2 summarizes key engineering considerations, including feed chemistry, sorbent format, contact mode, regeneration, product pathway, and process integration.

### 3.1 Scalable biomass production and harvesting for biosorption

Municipal wastewater, agricultural runoff and selected process waters may provide nutrient-rich media for photosynthetic biomass production while delivering water-polishing co-benefits. Raceway ponds remain a practical low-cost cultivation option in high-irradiance regions with available land because they are mechanically simple, energy efficient and scalable (Brennan and Owende, 2010; Bagchi et al., 2021).

Harvesting remains a major bottleneck. Low-energy primary concentration by settling, bioflocculation, or gravity-assisted separation should be prioritized, with secondary dewatering applied only when necessary (Uduman et al., 2010; Christenson and Sims, 2011). Because drying and lyophilization are major cost and energy drivers, industrial deployment should prioritize wet immobilized or partially dewatered biomass while reporting performance on a comparable dry-mass basis.

Affinity-based systems depend less on biomass volume than on binder production, purification, immobilization, and operational stability. Proteins and peptides must be assessed by expression yield, purification demand, support compatibility, regeneration stability, and lifetime, in addition to affinity and selectivity.

### 3.2 Leachate conditioning and solubility constraints

Industrial leachates are commonly acidic (pH 1–3). Under acidic sulfate and chloride conditions, many REEs remain soluble, whereas increasing pH can promote adsorption or precipitation as oxides, hydroxides, phosphates or carbonates (Jha et al., 2016; Binnemans et al., 2013). Conversely, very low pH suppresses biosorption through proton competition and can destabilize biological materials (Giese, 2020; Fomina and Gadd, 2014). The operating dilemma is therefore fundamental: the acidity that maintains REE solubility can also inhibit binding.

For living *G*. *sulphuraria*, sorption at its growth optimum of pH 1.5–2.5 is poor but can increase by up to 24-fold at pH 5.0 under phosphate-free conditions (Kastenhofer et al., 2024). Raising the pH simultaneously increases the risk of REE phosphate precipitation, which can occur above pH 2-3. Acid tolerance and sorption performance should therefore not be equated. *G. sulphuraria* grows across pH 0.5-4.0, but growth-optimal acidity does not imply optimal uptake. Conditioning should accordingly be limited to the minimum adjustment that preserves REE solubility while improving adsorption. Nevertheless, effective acidic recovery is possible. Immobilized *G*. *sulphuraria* removed 97.2% La^3+^, 96.2% Y^3+^ and 98.9% Sm^3+^ from REE-containing acid mining drainage (Sun et al., 2022).

Key development routes include phosphate-depleted conditioning, decoupling biomass production from capture by using inactivated biomass at an adjusted pH, and engineering acid-stable binders. In our view, resolving this trade-off is likely to advance industrial deployment more effectively than further incremental gains in equilibrium capacity.

### 3.3 Continuous operation, regeneration and product recovery

Industrial relevance cannot be established from batch removal experiments alone. Recovery systems should be evaluated in repeated-batch, continuous, or semi-continuous column configurations that allow breakthrough analysis, pressure-drop assessment, and staged regeneration (Gupta et al., 2019; Jin et al., 2017). Suitable formats may include packed or expanded beds, membranes, and particle-based systems.

Regeneration is central to economic and environmental viability. REEs should be desorbed by controlled elution that maximizes recovery while minimizing sorbent degradation. Assessment should include desorption efficiency, capacity and selectivity retention, material loss, and the composition and volume of the REE-enriched eluate. The recovered stream must be compatible with downstream precipitation, crystallization, solvent extraction, protein-based purification, or conversion to rare-earth oxides. The same principles apply to protein- and peptide-based affinity matrices.

## 4 Translational criteria for industrial deployment

Biological metal recovery systems must be evaluated under industrially relevant conditions before progressing beyond proof of concept. Core requirements include validation in real feeds, selectivity under realistic competitor-ion conditions, regeneration and reuse, process-relevant operation, and a defined downstream product route (Supplementary Table S3). High affinities or removal efficiencies measured in simplified synthetic solutions are important starting points, but they do not establish industrial feasibility.

### 4.1 Benchmarking in realistic matrices

Industrial relevance should be assessed using standardized performance metrics, rather than removal efficiencies alone. Supplementary Table S4 summarizes representative quantitative data for bioleaching, biosorption, bioaccumulation, and biologically derived nanomaterial adsorbents. Reported values range from adsorption capacities above 400 mg g^-1^ for selected nanomaterial-based adsorbents to REE recoveries above 90% for some phototrophs and 78% recovery from authentic post-mining waters using metabolically active extremophilic bacteria. Regeneration performance also varied widely. Some sorbents retain useful performance over multiple adsorption-desorption cycles, whereas lifecycle data remain unavailable for many systems. These values are not directly comparable because feed chemistry, pH, organism or material preparation, and calculation bases differ.

Accordingly, future studies should report adsorption capacity or loading capacity (mg g^-1^), recovery efficiency (%), distribution coefficients, separation factors, concentration factors, adsorption and desorption kinetics, regeneration stability, mass balance, eluate composition, and the concentrations of major competing ions such as Fe, Al, Ca and Mg (Jin et al., 2017; Dong et al., 2021; Brown et al., 2023). Initial and final concentrations of individual REEs, pH, ionic strength, sorbent format, and operating conditions should be reported. Validation should progress from single-element solutions to multicomponent synthetic leachates and ultimately to authentic industrial feeds with minimal pretreatment. Standardized benchmarking will support objective comparison and accelerate their translation into industrial hybrid bio(hydro)metallurgical processes.

### 4.2 Operational robustness and sorbent lifecycle

Operational constraints differ among biological platforms. Bioleaching and bioaccumulation require metabolically active organisms that tolerate acidity, salinity, and metal toxicity. Biosorbents require mechanical stability, low biomass loss, and sustained performance over repeated adsorption-desorption cycles. Protein and biomaterial systems additionally require stable immobilization, fouling resistance, and retention of selectivity after regeneration.

### 4.3 Systems-level integration and economic sustainability

Tailored biological systems are most promising within hybrid flowsheets, where they complement rather than replace established hydrometallurgy. Early techno-economic assessment (TEA) and life-cycle assessment (LCA) should determine whether biological recognition reduces reagent use, energy demand, waste generation, or process complexity sufficiently to offset the costs of biomass production, binder purification, immobilization, conditioning, and residue management (Jin et al., 2017; Schreiber et al., 2021; Vahidi and Zhao, 2017). Biological mobilization is most likely to add value where conventional processing is constrained by low grade, complex mineralogy, or poor accessibility.

Early adoption is therefore most plausible as modular, bolt-on enrichment or polishing units for dilute or chemically complex side streams (Jin et al., 2017; Dong et al., 2021). Regeneration and reuse represent critical determinants of economic feasibility. Biological sorbents and affinity materials should be evaluated on the basis of long-term retention of capacity, selectivity, mechanical integrity, and product quality, rather than initial performance alone (Gupta et al., 2019; Ye et al., 2024; Hussain et al., 2024). For cell-based systems, nutrient supply, biomass handling, and regeneration strongly influence cost. Nutrient-rich agricultural runoff or municipal wastewater may lower inputs while providing water-polishing benefits (Brennan and Owende, 2010; Christenson and Sims, 2011; Bagchi et al., 2021). For protein systems, expression, purification, immobilization and operational lifetime are likely to be the dominant cost drivers (Singh and Herzer, 2018; Dong et al., 2021).

An order-of-magnitude calculation illustrates the resulting material demand. One cubic meter of conditioned leachate containing 10 mg L^-1^ total REEs contains 10 g of recoverable metal. At a biosorption capacity of ∼70 mg g^-1^ (Paper et al., 2023b), about 140 g of dry biomass is required per loading cycle. A 100 m^3^ d^-1^ module would therefore require 14 kg dry biomass per pass, or roughly 1.4 kg d^-1^ of make-up material if the sorbent remains usable for ten cycles. At a protein-sorbent capacity of ∼1 mg REE g^-1^ (Ye et al., 2023), capturing the full 10 g would require on the order of 10 kg of functionalized material. Targeting only a smaller, high-value fraction would reduce this requirement proportionally. Upstream preconcentration therefore reduces volumetric load, contact time, and competitor-ion exposure, but not the binder inventory required for a given REE throughput. This supports the use of affinity modules as a final polishing step and identifies loading capacity, rather than affinity alone, as a critical scale-up parameter.

The strongest deployment case is consequently a modular recovery platform that extracts value from otherwise uneconomic side streams while reducing the chemical burden of conventional processing.

## 5 Key challenges and research needs

Despite rapid progress, tailored bio(hydro)metallurgy faces scientific and engineering challenges that must be resolved before industrial deployment.

### 5.1 Molecular and mechanistic refinement

A major limitation is the incomplete mechanistic understanding of biological sorbents, bioaccumulators, and molecular binders, particularly the relationship among feed chemistry, biomass composition, functional groups, and selectivity. FTIR, XPS, and synchrotron-based methods can resolve binding motifs and surface interactions (Fomina and Gadd, 2014; Cheng et al., 2019; Paper et al., 2023b; Plouviez et al., 2024). Structured databases linking biological composition, functional-group density, capacity, selectivity, and regeneration behavior would further support rational selection and engineering of recovery materials.

LanM and LanP exhibit exceptional group selectivity for REEs (Cotruvo et al., 2018; Dong et al., 2021; Hemmann et al., 2023), and metagenomic exploration is likely to reveal additional systems. Separation of adjacent lanthanides remains much more difficult. A metal-sensitive LanM dimer achieved single-stage column separation of Nd^3+^ and Dy^3+^ to greater than 98% individual-element purity by exploiting radius-dependent differences in dimerization affinity. The related chaperone LanD favors larger lanthanides during dimerization and has been proposed for tandem use with LanM to separate REE mixtures (Mattocks et al., 2023; Larrinaga et al., 2024). Future work should resolve lanthanide uptake, trafficking, storage and homeostasis and translate these insights into engineered proteins, peptides, and immobilized biomaterials.

Protein and peptide engineering must be linked to thermodynamic, kinetic, and process-level characterization. Studies should determine whether performance is limited by affinity, mass transfer, elution efficiency, fouling, protein degradation, or support failure under relevant conditions (Ye et al., 2024; Dong et al., 2021; Rodriguez et al., 2020; Hussain et al., 2024). High-affinity binders must also provide adequate loading capacity, defined binding and elution kinetics, and stable performance under flow. AI-guided structure prediction and design may accelerate the development of novel binders but require experimental validation in realistic matrices and over repeated regeneration cycles (Casadevall et al., 2023; Bennett et al., 2023).

### 5.2 Engineering for operational robustness

Translation requires operation in chemically variable feeds. High salt concentrations, competing metals, and organic matter can alter metal speciation, occupy binding sites, foul supports, and reduce selectivity (Fomina and Gadd, 2014; Giese, 2020; Brown et al., 2023). Immobilization must preserve binding function while providing mechanical integrity and enabling repeated regeneration.

Scale-up should progress from laboratory screening to pilot testing with real side streams. Key variables include feed variability, hydraulic residence time, breakthrough behavior, pressure drop, sorbent replacement, eluent recycling, residue handling, and integration as bolt-on modules within existing metallurgical facilities (Park et al., 2017; Jin et al., 2017).

### 5.3 Techno-economic and systems integration

Economic viability depends on site-specific trade-offs among recovery performance, cultivation and harvesting, conditioning, immobilization, binder production, regeneration, transport, and residue management. Feasibility studies should define system boundaries and quantify transport distances, material stability, quality-control requirements, regulatory constraints, secondary residues, potential sludge generation, and water-polishing co-benefits relative to conventional hydrometallurgy.

Federated open-access databases linking organism properties, omics data, matrix chemistry, binding performance, and process outcomes could support digital twins for predicting matrix interference and guiding the selection of strains or binders for specific feeds.

## 6 Outlook - toward precision biohydrometallurgy

Biological metal recovery is moving beyond classical biomining, which focused on bulk mobilization, toward precision biohydrometallurgy that combines molecular recognition, selective binding, and engineered biomaterials. The central question is no longer whether biological systems interact with REEs, but how these interactions can be engineered and integrated into economically credible flowsheets.

The emerging opportunity lies in dilute, heterogeneous, and chemically complex secondary streams. Conventional solvent extraction remains the industrial benchmark for concentrated feeds, whereas biological recognition provides a complementary separation principle for matrices in which low REE concentrations and abundant competitors undermine conventional economics. In a cascading hybrid flowsheet, microbial processes mobilize metals, biomass-based materials provide intermediate enrichment, and engineered proteins or biomaterials deliver high-resolution separation before conventional refining. This division of labor reduces the demands placed on each biological component and converts difficult feeds into smaller, cleaner, REE-enriched streams.

Progress now depends on systematic validation in authentic matrices, quantitative competitor-ion testing, regeneration and reuse, continuous-flow operation, and integrated TEA and LCA. Biological modules must be evaluated according to selectivity, throughput, stability, lifecycle, product quality, and compatibility with existing infrastructure. By combining biological precision with established metallurgy, precision biohydrometallurgy could extend recovery to previously uneconomic streams and strengthen the circularity and resilience of critical-minerals supply chains.

## Data Availability

The original contributions presented in the study are included in the article/Supplementary Material, further inquiries can be directed to the corresponding authors.
