# Supplementary material for: Towards precision bio(hydro)metallurgy: tailored biological systems for rare earth element recovery from dilute and secondary streams
Source: Front Bioeng Biotechnol. 2026 Oct 28;14:1920659. doi: 10.3389/fbioe.2026.1920659 (PMC13751488; doi:10.3389/fbioe.2026.1920659)
Supplement: Supplementary file 1 [file DataSheet1.pdf]

Supplementary Material

1 Supplementary Figures and Tables

1.1 Supplementary Figures

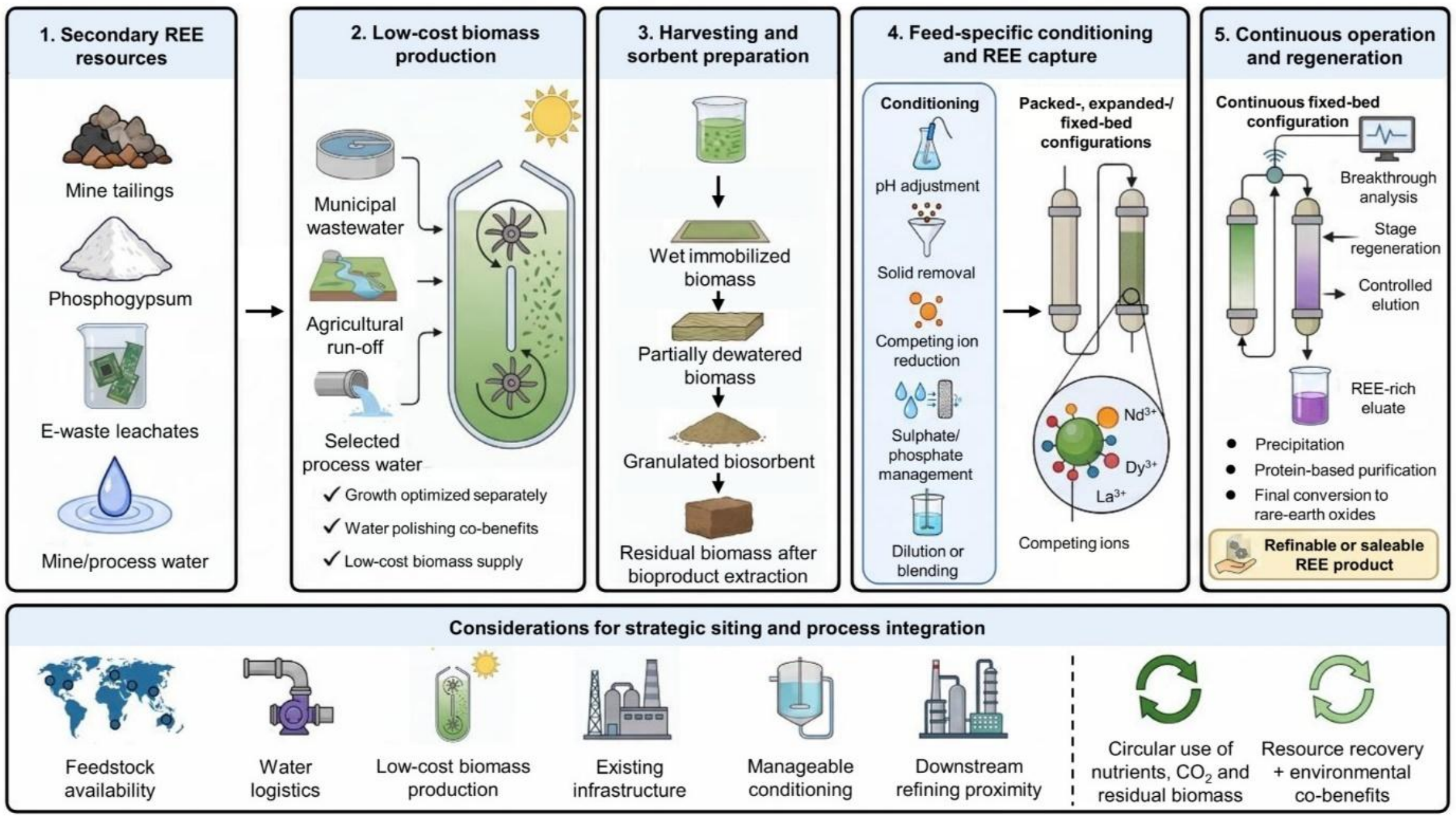

Supplementary Figure S1. From waste to resource: deployment opportunities for biological REE recovery. The scheme links dilute and heterogeneous REE-containing secondary streams to low-cost biomass production, sorbent preparation, feed-specific conditioning, continuous column operation, regeneration and product recovery. Strategic siting and process integration connect feedstock availability, water logistics, existing infrastructure, conditioning requirements, downstream refining, and circular resource use. Created in BioRender. Lübben, M. (2026). https://BioRender.com/2ok63hq

1.2 Supplementary Tables

Supplementary Table S1. Critical comparison of biological REE-recovery approaches and their positioning relative to conventional hydrometallurgy.

Process Stage	Selectivity	Operating Window	Key Advantages	Key Limitations	Best-Fit Scenario	Maturity	References
**Stage 1 – Solubilization**	Low; co-mobilizes REEs with Fe, Al and other matrix metals	pH ~1–3 (chemolithotrophs); moderate pH (fungal organic acids)	Acts directly on solid matrices; biologically generated lixiviant can reduce reagent input; builds on established biomining practice	Slow kinetics; limited selectivity; produces chemically complex leachates that require downstream cleanup	Low-grade solid residues, including tailings, red mud, phosphogypsum, coal discards, and e-waste	**Pilot for REEs; commercial for base metals**	(Rawlings and Johnson 2007; Rasoulnia et al. 2021; Brown et al. 2023; Soto-Montandon et al. 2025)
**Stage 2 – Biosorption**	REE-group enrichment relative to base metals; limited inter-REE selectivity	pH ~4–7 (mildly acidic to near-neutral)	Fast and metabolism-independent; compatible with low-cost, immobilizable biomass; regenerable; robust to feed variability	Limited inter-lanthanide resolution; finite capacity; proton competition, fouling, and sorbent degradation at low pH	Dilute, conditioned leachates and wastewaters; volume reduction before final polishing	**Bench–pilot**	(Fomina and Gadd 2014; Gupta et al. 2019; Giese 2020; Sun et al. 2022; Paper et al. 2023b)
**Stage 2 – Bioaccumulation**	Moderate; species- and metabolism-dependent	Near-neutral; requires viable, active cells	Can concentrate metals beyond surface-site capacity and can be combined with water polishing	Requires living cells; slower than biosorption; constrained by metal-toxicity; recovery and regeneration are more difficult	Low-toxicity dilute streams in which biomass or water treatment provides a co-benefit	**Laboratory**	(Jin et al. 2017; Singh et al. 2023; Rocha et al. 2024; Vítová and Mezricky 2024)
**Stage 3 – Affinity separation**	Highest; inter-lanthanide (single-stage Nd/Dy separation to >98% purity)	Near-neutral; pre-cleaned, low-solids eluate	Molecular recognition; tunable toward target element pairs; compatible with metabolism-free operation and single-stage separation	Binder production cost; immobilization, fouling, loading capacity and long-term stability remain unproven at scale	Pre-enriched eluates requiring high-resolution purification or a final product cut	**Laboratory–early bench**	(Cotruvo et al. 2018; Dong et al. 2021; Mattocks et al. 2023; Ye et al. 2023; Hussain et al. 2024)
Conventional solvent extraction *(benchmark)*	*High after many sequential stages*	*Strongly acidic*	*Mature, high-throughput, and proven at industrial scale*	*Typically requires 100 to more than 1000 separation stages; high solvent, acid and energy demand; economics deteriorate sharply at low grade; large waste volumes*	*High-grade, concentrated primary feeds*	***Industrial standard***	(Binnemans et al. 2013; Jha et al. 2016; Vahidi and Zhao 2017; Geng et al. 2023)

Together, the stages form a selectivity–maturity cascade: low-selectivity mobilization feeds REE-group enrichment, which supplies cleaner eluates to affinity separation. Conventional solvent extraction remains the benchmark for high-grade, concentrated feeds, whereas the biological cascade targets dilute, heterogeneous secondary resources for which conventional selectivity and economics deteriorate.

Supplementary Table S2. Practical engineering considerations for biological REE recovery modules.

Engineering parameter	Relevance	Key constraints/Reporting needs	References
Feed chemistry and pH window	Controls speciation, proton competition and apparent capacity.	Report feed origin, pH, individual REEs, major competitors, organics, and solids; conditioning is often required for acidic leachates.	Paper et al. 2023b; Giese 2020; Heilmann et al. 2021
Functional groups and biosorbent chemistry	Governs binding mechanism, capacity, and selectivity.	Carboxyl, hydroxyl, phosphate, sulfate and EPS-associated sites often contribute; characterize biomass chemistry rather than assuming a universal sorbent.	Philippis et al. 2011; Fomina and Gadd 2014; Paper et al. 2023a
Kinetics and contact mode	Determines reactor sizing, throughput, and mass-transfer performance.	Report uptake kinetics, solid-to-liquid ratio, residence time, flow rate and mass-transfer limitations.	Heilmann et al. 2021; Paper et al. 2023b
Regeneration, desorption, and durability	Core requirement for cost, reuse and circularity.	Report eluent chemistry and volume, desorption efficiency, cycle number, capacity and selectivity retention, material loss, and eluate concentration.	Hussain et al. 2024; Giese 2020; Sun et al. 2022; Ye et al. 2023
Format and integration	Batch performance alone does not demonstrate process feasibility.	Test repeated-batch, packed-bed, expanded-bed, membrane, or particle-based formats; report breakthrough behavior and pressure drop where relevant.	Park et al. 2017; Jin et al. 2017; Dong et al. 2021; Hussain et al. 2024
Product pathway and purity target	Links biological capture to a recoverable and refinable product.	Define the intended output: REE-enriched eluate, mixed concentrate, or purified fraction; report impurity profile and downstream recovery route.	Hussain et al. 2024; Giese 2020; Jha et al. 2016; Binnemans et al. 2013
Scalability and siting	Determines whether deployment is logistically and economically credible.	Evaluate residue availability, biomass or binder production, water and energy logistics, existing infrastructure, and access to downstream refining.	Jin et al. 2017; Schreiber et al. 2021; Brown et al. 2023
Sustainability and safety	Biological origin does not guarantee lower environmental impact.	Include TEA/LCA, reagent and energy demand, residues, water use, and safety considerations such as spores, genetically modified organisms, and spent sorbents.	Brown et al. 2023; Schreiber et al. 2021; Vahidi and Zhao 2017; Ye et al. 2024

Engineering requirements are specific to the process-function. Solubilization modules must be evaluated by mobilization and leachate quality, enrichment modules by concentration performance and regeneration, and affinity modules by selectivity, binder lifetime and product purity. Across all modules, realistic matrices and repeated operation are essential.

Supplementary Table S3. Minimum translational criteria for moving biological REE recovery toward pilot and industrial deployment.

Criterion	Minimum information to report	Laboratory proof-of-concept	Industrial relevance	References
Real-feed validation	Feed origin, pH, individual/total REEs, major competitors, salinity, organics and suspended solids.	Use authentic leachates/wastewaters, not only single-salt lab solutions.	Feed variability, conditioning demand and impurity load before flowsheet integration.	Jha et al. 2016; Brown et al. 2023; Singh et al. 2023; Náhlík et al. 2023
Core adsorption dataset	Biomass/binder state, preparation, dry-mass basis, capacity, recovery, distribution coefficient, separation factor, concentration factor, kinetics and mass balance.	Report biomass preparation, calculation basis, and a complete mass balance.	Enables reproducible comparison across solubilization, enrichment, and affinity modules and distinguishes recovery from non-selective removal.	Fomina and Gadd 2014; Giese 2020; Gupta et al. 2019
Selectivity under competitors	Target REEs, competing ions, salts and organic matter; report enrichment and product purity, not removal alone.	Single-ion tests identify potential but should not be overinterpreted.	Confirms whether selectivity persists at feed-relevant competitor ratios.	Cotruvo et al. 2018; Dong et al. 2021; Ye et al. 2023; Ye et al. 2024
Regeneration and reuse	Eluent chemistry and volume, desorption efficiency, retention of capacity and selectivity, and material loss.	Demonstrate recoverability and the first reuse cycles.	Determines viability through stable multi-cycle performance and generation of concentrated eluate.	Ye et al. 2023; Sun et al. 2022; Giese 2020
Process-relevant operation mode	Flow rate, residence time, breakthrough, pressure drop, column dimensions and regeneration strategy.	Batch tests are sufficient only for early screening.	Provides continuous or semicontinuous design data for scale-up and integration.	Jin et al. 2017; Dong et al. 2021; Ye et al. 2023
Defined downstream product route	Eluate concentration, impurity profile, precipitation or polishing route, and final product purity.	Demonstrate that REEs can be eluted or otherwise recovered from the biological material.	Links biological capture to a marketable or refinable REE product.	Binnemans et al. 2013; Jha et al. 2016
Sustainability screen	Material lifetime, biomass/binder production, harvesting/drying, immobilization, reagents, energy, water, residues and TEA/LCA boundaries.	Use a cautious qualitative assessment and a complete mass balance.	Identifies cost, energy, reagent, water, and residue hotspots; biological origin alone is insufficient.	Fomina and Gadd 2014; Jin et al. 2017; Vahidi and Zhao 2017; Schreiber et al. 2021
Regulatory and safety considerations	GMO status, containment, leachate toxicity, spore and operator safety, spent biomass and residue classification.	Identify potential hazards.	Defines permitting, containment, operator-safety, social acceptance and spent-sorbent requirements.	Brown et al. 2023; Ye et al. 2024

Minimum translational criteria should be reported according to process function. A biological system should not be judged only by high uptake or high leaching efficiency in simplified media. To support scale-up, studies should demonstrate performance in realistic matrices, repeated operation, regeneration, product recovery and credible integration into a hybrid metallurgical flowsheet.

Supplementary Table S4. Representative quantitative performance metrics reported for biological REE recovery systems.

Technology	Biological system	Feed	Representative performance	Regeneration	Reference
Adsorption using microbially synthesized nanomaterials	Microorganism-mediated nanomaterials and nanoparticles	Rare earth mine wastewater containing REEs	99% Eu^{3+} adsorption (Fe_3O_4@HA magnetic NPs); 86 mg g^{-1} Gd^{3+} (IIP-CS/CNT–SiO_2@Fe_3O_4); 22.0 mg g^{-1} La^{3+} (MSCA-12); 430.4 mg g^{-1} Dy^{3+} and 281.1 mg g^{-1} Sm^{3+} (ZIF-8 NPs)	3–18 adsorption-desorption cycles across the summarized studies, depending on adsorbent	Yan et al. 2025
Biosorption	Dead or metabolically inactive microbial biomass (algae, bacteria, fungi, yeasts)	REE-containing aqueous solutions, industrial wastewater, leachates, acid mine drainage, e-waste and clay-mineral leachates, mining effluents	*Chlamydomonas reinhardtii* – La^{3+} 1.03 mmol g^{-1}; *Turbinaria conoides* – La^{3+} 1.11, Ce^{3+} 1.09, Eu^{3+} 0.91, Yb^{3+} 0.70 mmol g^{-1}; *Chlorella vulgaris* – Nd^{3+} 157.21 mg g^{-1}; *Bacillus thuringiensis* – Eu^{3+} 160 mg g^{-1}; *Saccharomyces cerevisiae* – La^{3+} 70–80 mg g^{-1}; *Bacillus subtilis*/alginate – La^{3+} 81.10, Sm^{3+} 83.51 mg g^{-1}	Not reported	Giese 2020
Bioleaching, biosorption and bioaccumulation	Microalgae, macroalgae, cyanobacteria, bacteria, fungi and yeasts	REE ores (monazite, bastnäsite, apatite), WEEE, phosphogypsum, red mud, fly ash, industrial effluents, acid mine drainage, luminophores	>90% recovery of Nd^{3+}, Dy^{3+}, La^{3+} by *Galdieria sulphuraria* (0.5 ppm); 70% recovery of Yb^{3+}, Ce^{3+}, Nd^{3+}, Eu^{3+}, La^{3+} by *Gracilaria gracilis* (0.5 mg L^{-1}); 83% Ce^{3+} by *Nannochloropsis oculata*; 27.3 mg kg^{-1} day^{-1} REE accumulation by *Desmodesmus quadricauda* on red mud	Not reported	Vítová and Mezricky 2024
Biosynthesized nanocomposite adsorption	BC@FeNPs-EPS derived from *Bacillus cereus* (EPS-coated Fe nanoparticles)	Rare earth mine wastewater (Eu^{3+}, Tb^{3+}, Ce^{3+})	Eu^{3+} 90.2% removal (synthetic), 88% (real mine wastewater); Tb^{3+} 89%; Ce^{3+} 89%; q_{max} = 23.39 mg g^{-1} for Eu^{3+}	Desorbed with ultrapure water; Five cycles: Eu^{3+} removal 89, 89, 81, 73, 69%	Yan et al. 2023
Bioaccumulation (selective microbial uptake)	Live extremophilic methanotroph *Methylacidiphilum fumariolicum* SolV	Low-grade REE sources: post-mining and industrial decomposition waters	78% total REE removal from Königstein water over 6 cycles (1145→253 nM); 69% from artificial Waste Mix over 6 cycles (607→187 nM); 47.4% of total lanthanides removed in a 3.7 L bioreactor	Biomass removed by centrifugation each cycle, fresh SolV added; demonstrated over 6 sequential cycles	Singer et al. 2023

Values are reported as presented in the cited sources and are not directly comparable because feed composition, pH, biological system, material preparation, analytical basis, and performance definitions differ.

2 References

Binnemans, Koen/Peter Tom Jones/Bart Blanpain/Tom Van Gerven/Yongxiang Yang/Allan Walton/Matthias Buchert (2013). Recycling of rare earths: a critical review. Journal of Cleaner Production 51, 1–22. https://doi.org/10.1016/j.jclepro.2012.12.037.

Brown, Rebecca M./Amin Mirkouei/David Reed/Vicki Thompson (2023). Current nature-based biological practices for rare earth elements extraction and recovery: Bioleaching and biosorption. Renewable and Sustainable Energy Reviews 173, 113099. https://doi.org/10.1016/j.rser.2022.113099.

Cotruvo, Joseph A./Featherston, Emily R./Mattocks, Joseph A./Ho, Jackson V./Laremore, Tatiana N. (2018). Lanmodulin: A Highly Selective Lanthanide-Binding Protein from a Lanthanide-Utilizing Bacterium. Journal of the American Chemical Society 140 (44), 15056–15061. https://doi.org/10.1021/jacs.8b09842.

Dong, Ziye/Mattocks, Joseph A./Deblonde, Gauthier J-P/Hu, Dehong/Jiao, Yongqin/Cotruvo, Joseph A./Park, Dan M. (2021). Bridging Hydrometallurgy and Biochemistry: A Protein-Based Process for Recovery and Separation of Rare Earth Elements. ACS central science 7 (11), 1798–1808. https://doi.org/10.1021/acscentsci.1c00724.

Fomina, Marina/Gadd, Geoffrey Michael (2014). Biosorption: current perspectives on concept, definition and application. Bioresource technology 160, 3–14. https://doi.org/10.1016/j.biortech.2013.12.102.

Geng, Yong/Sarkis, Joseph/Bleischwitz, Raimund (2023). How to build a circular economy for rare-earth elements. Nature 619 (7969), 248–251. https://doi.org/10.1038/d41586-023-02153-z.

Giese, Ellen Cristine (2020). Biosorption as green technology for the recovery and separation of rare earth elements. World journal of microbiology & biotechnology 36 (4), 52. https://doi.org/10.1007/s11274-020-02821-6.

Gupta, Nishesh Kumar/Gupta, Anjali/Ramteke, Prajakta/Sahoo, Harekrushna/Sengupta, Arijit (2019). Biosorption-a green method for the preconcentration of rare earth elements (REEs) from waste solutions: A review. Journal of Molecular Liquids 274, 148–164. https://doi.org/10.1016/j.molliq.2018.10.134.

Heilmann, Marcus/Breiter, Roman/Becker, Anna Maria (2021). Towards rare earth element recovery from wastewaters: biosorption using phototrophic organisms. Applied microbiology and biotechnology 105 (12), 5229–5239. https://doi.org/10.1007/s00253-021-11386-9.

Hussain, Zohaib/Dwivedi, Divya/Kwon, Inchan (2024). Recovery of rare earth elements from low-grade coal fly ash using a recyclable protein biosorbent. Frontiers in bioengineering and biotechnology 12, 1385845. https://doi.org/10.3389/fbioe.2024.1385845.

Jha, Manis Kumar/Kumari, Archana/Panda, Rekha/Rajesh Kumar, Jyothi/Yoo, Kyoungkeun/Lee, Jin Young (2016). Review on hydrometallurgical recovery of rare earth metals. Hydrometallurgy 161, 77. https://doi.org/10.1016/j.hydromet.2016.01.003.

Jin, Hongyue/Park, Dan M./Gupta, Mayank/Brewer, Aaron W./Ho, Lewis/Singer, Suzanne L./Bourcier, William L./Woods, Sam/Reed, David W./Lammers, Laura N./Sutherland, John W./Jiao, Yongqin (2017). Techno-economic Assessment for Integrating Biosorption into Rare Earth Recovery Process. ACS Sustainable Chemistry & Engineering 5 (11), 10148–10155. https://doi.org/10.1021/acssuschemeng.7b02147.

Mattocks, Joseph A./Jung, Jonathan J./Lin, Chi-Yun/Dong, Ziye/Yennawar, Neela H./Featherston, Emily R./Kang-Yun, Christina S./Hamilton, Timothy A./Park, Dan M./Boal, Amie K./Cotruvo, Joseph A. (2023). Enhanced rare-earth separation with a metal-sensitive lanmodulin dimer. Nature 618 (7963), 87–93. https://doi.org/10.1038/s41586-023-05945-5.

Náhlík, Vít/Čížková, Mária/Singh, Anjali/Mezricky, Dana/Rucki, Marian/Andresen, Elisa/Vítová, Milada (2023). Growth of the Red Alga Galdieria sulphuraria in Red Mud-Containing Medium and Accumulation of Rare Earth Elements. Waste and Biomass Valorization 14 (7), 2179–2189. https://doi.org/10.1007/s12649-022-02021-3.

Paper, Michael/Jung, Patrick/Koch, Max/Lakatos, Michael/Nilges, Tom/Brück, Thomas B. (2023a). Stripped: contribution of cyanobacterial extracellular polymeric substances to the adsorption of rare earth elements from aqueous solutions. Frontiers in bioengineering and biotechnology 11, 1299349. https://doi.org/10.3389/fbioe.2023.1299349.

Paper, Michael/Koch, Max/Jung, Patrick/Lakatos, Michael/Nilges, Tom/Brück, Thomas B. (2023b). Rare earths stick to rare cyanobacteria: Future potential for bioremediation and recovery of rare earth elements. Frontiers in bioengineering and biotechnology 11, 1130939. https://doi.org/10.3389/fbioe.2023.1130939.

Park, Dan M./Brewer, Aaron/Reed, David W./Lammers, Laura N./Jiao, Yongqin (2017). Recovery of Rare Earth Elements from Low-Grade Feedstock Leachates Using Engineered Bacteria. Environmental science & technology 51 (22), 13471–13480. https://doi.org/10.1021/acs.est.7b02414.

Philippis, Roberto de/Colica, Giovanni/Micheletti, Ernesto (2011). Exopolysaccharide-producing cyanobacteria in heavy metal removal from water: molecular basis and practical applicability of the biosorption process. Applied microbiology and biotechnology 92 (4), 697–708. https://doi.org/10.1007/s00253-011-3601-z.

Rasoulnia, Payam/Barthen, Robert/Lakaniemi, Aino-Maija (2021). A critical review of bioleaching of rare earth elements: The mechanisms and effect of process parameters. Critical Reviews in Environmental Science and Technology 51 (4), 378–427. https://doi.org/10.1080/10643389.2020.1727718.

Rawlings, Douglas E./Johnson, D. Barrie (2007). The microbiology of biomining: development and optimization of mineral-oxidizing microbial consortia. Microbiology (Reading, England) 153 (Pt 2), 315–324. https://doi.org/10.1099/mic.0.2006/001206-0.

Rocha, Raquel A./Alexandrov, Kirill/Scott, Colin (2024). Rare earth elements in biology: From biochemical curiosity to solutions for extractive industries. Microbial biotechnology 17 (6), e14503. https://doi.org/10.1111/1751-7915.14503.

Schreiber, Andrea/Marx, Josefine/Zapp, Petra (2021). Life Cycle Assessment studies of rare earths production - Findings from a systematic review. The Science of the total environment 791, 148257. https://doi.org/10.1016/j.scitotenv.2021.148257.

Singer, Helena/Steudtner, Robin/Sottorff, Ignacio/Drobot, Björn/Pol, Arjan/Op den Camp, Huub J. M./Daumann, Lena J. (2023). Learning from nature: recovery of rare earth elements by the extremophilic bacterium Methylacidiphilum fumariolicum. Chemical Communications 59 (59), 9066–9069. https://doi.org/10.1039/d3cc01341c.

Singh, Anjali/Čížková, Mária/Náhlík, Vít/Mezricky, Dana/Schild, Dominik/Rucki, Marian/Vítová, Milada (2023). Bio-removal of rare earth elements from hazardous industrial waste of CFL bulbs by the extremophile red alga Galdieria sulphuraria. Frontiers in microbiology 14, 1130848. https://doi.org/10.3389/fmicb.2023.1130848.

Soto-Montandon, Fernanda/Gillane, Rosemary/Marcellin, Esteban/Harrison, Susan T. L./Villa-Gomez, Denys (2025). Bioleaching for critical metal recovery from bauxite residue- unlocking waste valorization. Frontiers in bioengineering and biotechnology Volume 13 - 2025. https://doi.org/10.3389/fbioe.2025.1685819.

Sun, Yabo/Lu, Tao/Pan, Yali/Shi, Menghan/Ding, Dan/Ma, Zhiwen/Liu, Jiuyi/Yuan, Yupeng/Fei, Ling/Sun, Yingqiang (2022). Recovering rare earth elements via immobilized red algae from ammonium-rich wastewater. Environmental science and ecotechnology 12, 100204. https://doi.org/10.1016/j.ese.2022.100204.

Vahidi, Ehsan/Zhao, Fu (2017). Environmental life cycle assessment on the separation of rare earth oxides through solvent extraction. Journal of Environmental Management 203, 255–263. https://doi.org/10.1016/j.jenvman.2017.07.076.

Vítová, Milada/Mezricky, Dana (2024). Microbial recovery of rare earth elements from various waste sources: a mini review with emphasis on microalgae. World journal of microbiology & biotechnology 40 (6), 189. https://doi.org/10.1007/s11274-024-03974-4.

Yan, Qiuting/Yang, Yalin/Chen, Wei/Weng, Xiulan/Owens, Gary/Chen, Zuliang (2023). Recovery and removal of rare earth elements from mine wastewater using synthesized bio-nanoparticles derived from Bacillus cereus. Chemical Engineering Journal 459, 141585. https://doi.org/10.1016/j.cej.2023.141585.

Yan, Qiuting/Yang, Zhiman/Chen, Zuliang (2025). Recent advances in microbial nanomaterials/nanoparticles synthesis and rare earth elements recovery from rare earth mine wastewater: A review. Chemical Engineering Journal 510, 161647. https://doi.org/10.1016/j.cej.2025.161647.

Ye, Quanhui/Jin, Xiuyu/Zhu, Baotong/Gao, Haifeng/Wei, Na (2023). Lanmodulin-Functionalized Magnetic Nanoparticles as a Highly Selective Biosorbent for Recovery of Rare Earth Elements. Environmental science & technology 57 (10), 4276–4285. https://doi.org/10.1021/acs.est.2c08971.

Ye, Quanhui/Wang, Dong/Wei, Na (2024). Engineering biomaterials for the recovery of rare earth elements. Trends in biotechnology 42 (5), 575–590. https://doi.org/10.1016/j.tibtech.2023.10.011.
